# Academic trajectory of Prof. Ana Marlúcia Oliveira: contributions to the field of food and nutrition in Brazil

**DOI:** 10.1590/1980-549720230042.2

**Published:** 2023-10-09

**Authors:** Rita de Cássia Ribeiro-Silva, Priscila Ribas de Farias Costa, Maria Ester Pereira da Conceição, Lucivalda Pereira Magalhães de Oliveira, Maria da Purificação Nazaré Araújo, Mônica Leila Portela de Santana, Nedja Silva dos Santos, Sandra Maria Chaves dos Santos, Valterlinda Alves de Oliveira Queiroz, Nilce de Oliveira, Pedro Israel Cabral de Lira, Mauricio Lima Barreto

**Affiliations:** Universidade Federal da Bahia, Escola de Nutrição – Salvador (BA), Brasil.; Universidade Federal de Pernambuco, Centro de Ciências da Saúde, Departamento de Nutrição – Recife (PE), Brasil.; Fundação Oswaldo Cruz, Instituto Gonçalo Moniz, Centro de Integração de Dados e Conhecimentos para Saúde – Salvador (BA), Brasil.

**Keywords:** Nutritional sciences, Biography, Faculty, Ciências da nutrição, Biografia, Docente

## Abstract

It will be presented the main academic contributions of Professor Ana Marlúcia Oliveira (AMO) (in memoriam), nutritionist, professor at the School of Nutrition at the Federal University of Bahia, Ph.D. in epidemiology and CNPQ Researcher level A, from 1980 to 2021. Professor Ana accumulated, throughout her academic career, scientific articles published in national and international journals; book and book chapters authored by her; papers presented at scientific events, in addition to guiding scientific projects, dissertations and theses. She has coordinated several research projects in the field of food and nutrition in public health, with a focus on nutritional epidemiology. The scope of the subjects addressed in her scientific production expressed the concern that mobilized her around the production of knowledge to face the complex health and nutrition problems in Brazil. Her way of being in the world, welcoming and caring for people who approached her seeking qualification opportunities, her example, words and teachings influenced, and still influence, the trajectory and training of nutritionists, professors and researchers at ENUFBA and other national and international institutions. She was a Brazilian researcher and intellectual committed to the health of the most vulnerable populations and the fight against malnutrition and hunger in our country. Her wide and fruitful work left us a legacy to be remembered and continued. Some of her friends, colleagues and collaborators pay this tribute to her memory, to her example and to the legacy she left for all of us and future generations.

## RESUMO

São apresentadas neste artigo as principais contribuições acadêmicas da Profa. Marlúcia Oliveira (AMO) (*in memoriam*), nutricionista, docente da Escola de Nutrição da Universidade Federal da Bahia, Doutora em Epidemiologia e Pesquisadora A do Conselho Nacional de Desenvolvimento Científico e Tecnológico (CNPQ), no período de 1980 a 2021. A Profa. Ana acumulou, ao longo de sua carreira acadêmica, artigos científicos publicados em periódicos nacionais e internacionais; livro e capítulos de livro de sua autoria; trabalhos apresentados em eventos científicos, além de ter orientado projetos de iniciação científica, dissertações e teses. Coordenou vários projetos de pesquisa no campo da alimentação e nutrição em saúde coletiva, com foco na epidemiologia nutricional. O escopo dos assuntos abordados na sua produção científica expressou a inquietude que a mobilizava em torno da produção de conhecimentos para o enfrentamento dos complexos problemas de saúde e nutrição do Brasil. Sua forma de ser no mundo, acolhendo e cuidando das pessoas que se aproximavam em busca de oportunidades de qualificação, seu exemplo, suas palavras e ensinamentos influenciaram, e ainda influenciam, a trajetória e a formação de nutricionistas e de professores e pesquisadores na Escola de Nutrição da Universidade Federal da Bahia (ENUFBA) e em outras instituições nacionais e internacionais. Trata-se de uma pesquisadora e intelectual brasileira comprometida com a saúde das populações mais vulneráveis e com o combate contra a desnutrição e a fome no nosso país. Sua vasta e fecunda obra nos deixou um legado a ser lembrado e continuado. Alguns dos seus amigos, colegas e colaboradores prestam este tributo à sua memória e ao legado que ela deixou para todos nós e para as próximas gerações.

## INTRODUCTION

This article aimed to present the academic trajectory of Prof. Ana Marlúcia Oliveira (in memoriam) and her important contributions to the field of food and nutrition and to the fight against hunger in Brazil. Prof. Ana Marlúcia Oliveira was a member of the School of Nutrition at Universidade Federal da Bahia (ENUFBA). Through this material, in addition to systematizing at least part of the professor’s contributions to teaching and research in food and nutrition in Bahia and Brazil, we are retracing part of the elements that make up the trajectory of the very conformation of this field of knowledge and practices, insofar as it was an active agent in this path. In her 45 years as a nutritionist, 42 of which teaching and training new professionals, she has always been at the heart of the most relevant theoretical and methodological debates, as will be presented in this text. Let us start by presenting a little bit of Professor Ana Marlúcia’s biography, who was born in the countryside of Bahia, in Itabuna, in 1949. She graduated in nutrition from *Universidade Federal da Bahia* (UFBA) in 1974, therefore, at a time of strong repression by the military dictatorship. In 1977, in those very difficult times, she left to undertake a master’s degree at the School of Public Health in Mexico, defending a dissertation whose theme would mark her searches as a researcher and academic activist: the epidemiology of malnutrition in Latin America. In 1980, she took over as a professor at ENUFBA, from which she retired in 2020; but she continued guiding and researching until the moment her health no longer allowed it. In her trajectory, she focused her studies on the social determinants of malnutrition in the different life cycles, particularly in childhood. The maternal-infant group was one of her passions, and she directed several studies and training of nutritionists, answering for years for the discipline of “Maternal-Infant Nutrition”. Ana had a restless soul and mind, of a researcher outside her comfort zone, which allowed her to dialogue with scholars from various areas of knowledge without losing focus: nutritional epidemiology and the health and nutrition of the most vulnerable groups in Brazilian society.

This record was organized into the main themes of Ana’s work and central aspects of her academic life: research, extension, and teaching. In times of limited inclusion of the poorest people in a public university, Ana achieved this goal and formed a whole family. For this reason and for the extended family she put together with her friends, students, and advisees, this article does not intend to be an act of elevation of her academic trajectory. In this country, where research in social nutrition continues to be essential, an invitation is made to young nutritionists and other researchers so that, in their training and practice, in services and in academia, they do not decline to pursue the path trodden by Prof. Ana, of a scholar committed to the fight for health and food as fundamental rights.

### From denunciations of hunger, malnutrition, and inequalities to prospecting alternatives for coping with the situation

**Figure 1.**
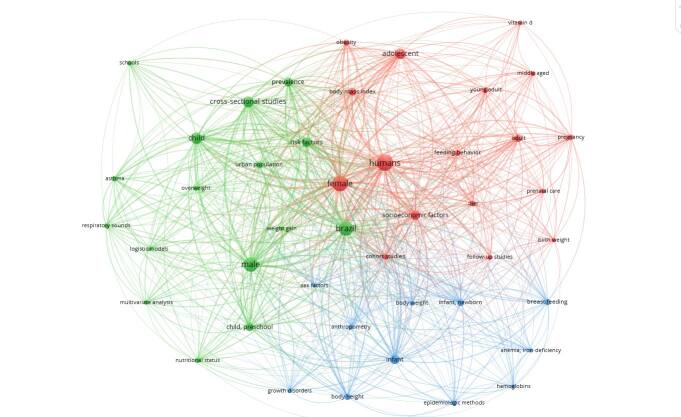
Bibliometric analysis — mapping of relevant themes (1994–2021).

Ana’s curriculum contains 124 published scientific articles. Through bibliometric analysis, it was possible to compose a thematic overview of her production (Figure 1). The identified terms reflect the concern and contemporary potential outlined in the field of food and nutrition from the perspective of collective health. The central focus of her academic production was to generate knowledge about the social determinants of complex existing or potential nutrition and health problems, which especially afflict mothers and children. This life cycle was Ana’s “baby”. In the subfield of Nutritional Epidemiology, which encompasses the entire range of nutritional diseases related to lack and/or excess of nutrients, Ana stood out, initially, with her studies focused on the identification of factors associated with nutritional deficiencies in the mother-child binomial, especially those of iron and vitamin A that plagued these groups in the initial period of her studies. Subsequently, accompanying the nutritional transition in the Brazilian population, her studies focused on childhood obesity and its determinants stand out.

Her first manuscripts contributed to the generation of knowledge aimed at understanding how social, political, and environmental determinants were associated with childhood diseases linked to poverty and hunger and how they were related to one another (protein-calorie malnutrition, nutritional deficiencies, infectious and parasitic diseases). Ana was a pioneer in establishing the idea that “hunger was a political issue”^1^. Understanding, for example, the impact of diarrhea on children’s nutritional status was a topic widely explored in her studies in the years 2000^2,3^. Over the years, great transformations occurred in various spheres of life in Brazilian society, accompanying what was happening across the globe, especially in the political, economic, social, cultural, and environmental fields, which constitute the main explanatory factors of the significant improvements in the health conditions observed^4^. The epidemiological scenario has undergone important changes. With the real increase in the minimum wage, improvements in education, government policies of social protection and other pro-equity public policies implemented after democratization and, more intensely, from the first Lula administration, malnutrition and childhood mortality declined sharply and life expectancy increased with more quality^5^. Thus, in the field of nutrition and health, the country faced new and difficult challenges. For example, in the last decade, the obesity rate in the country almost doubled, and the share of overweight people practically tripled^6^. Simultaneously, the growing availability of processed foods, high in calories and poor in nutrients, generated a new type of malnutrition, characterized by overweight people and, at the same time, precarious nutritional status (Brazilian nutritional paradox)^7,8^. Faced with this new epidemiological situation, Prof. Ana sought to formulate new hypotheses and study various strategies to show how much the procedures and interventions practiced (promotion of healthy practices and habits) could be efficient or effective for the promotion of health and a healthy weight; now exploring the theme in children and adolescents^9^. This change would be reflected in Ana’s research and publications, which began to explore themes related to obesity and food-related diseases, especially among adolescents, a group in which food choices are strongly influenced by social and environmental issues^10^.

With the advent of the COVID-19 pandemic, she was one of the first researchers to explore the topic in the field of food and nutritional security^11^. However, in the context of the Brazilian epidemiological transition, the emergence of modern diseases (for example, eating disorders and food-related illnesses) did not distance her from research on the epidemiology of the diseases of the most vulnerable, with an emphasis on factors of environmental, social, and political origin.

### Research and extension as intention

Consistent with her social responsibility and ideological option of working to strengthen the Public University, which must maintain its commitment to being free, of quality, inclusive and, therefore, socially and culturally referenced, Ana could not fail to dedicate herself to the extensionist role. The University Extension model to which he was enthusiastically linked was especially consolidated through Community Development Support projects, carried out in urban peripheries and rural communities, especially in municipalities marked by extreme poverty and consequently low social and nutrition and health indicators, both in Bahia and in other Northeastern states. In the latter case, through the Northeast Food and Nutrition Collaborating Center II (*Centro Colaborador em Alimentação e Nutrição Nordeste II* – CECAN NE II). These were projects aimed at improving the living conditions of families, carried out based on participatory work (families, workers, community representative entities, leaders, and the university) and also relying on some collaboration from public services. Eminently interdisciplinary projects, with a focus on sustainability and integrated with teaching and research, all of which generated publications, important in disseminating, especially to the university environment, the achievements of the extension effort. Ana was a member of the teaching teams, coordinated, updated and created intervention models that resulted in an improvement in the food and nutritional security framework with actions to feed malnourished children and innumerable technologies and educational practices in health care. In addition to the areas of health, nutrition and food, the projects included access to drinking water (especially in the semi-arid region), support for productive activities and the consequent generation of income, improvement of housing, training in various areas, with emphasis on Community Health Agents. It is also worth emphasizing that health managers, municipal technical teams, mayors of the municipalities were also subjects of attention in the projects, to receive guidance on how to implement and improve public policies, thus expanding the scope of those directly involved with local development. Due to the volume of actions carried out, duration, size and diversity of the teams and particularly for the good results achieved, it is possible to highlight the Cansanção Project^12^, the Integrated Rural Development Project of Sapeaçu (*Projeto de Desenvolvimento Rural Integrado de Sapeaçu* – PDRI)^13^, and the Support Project for the Development of the Municipal Food and Nutrition Security System in Mutuípe^10^, three municipalities in Bahia characterized by a high degree of economic and social vulnerability.

### Training of professionals, teachers, and researchers as a vocation

In teaching, Professor Ana made countless contributions. She marked the lives of generations of students, future professionals and in the different spaces of teaching and learning, she imprinted her way of training nutritionists to work in maternal and child care with an emphasis on the social determinants of diseases. Classrooms with blackboards, “secret” notebook with class notes (later transparencies, slides, and multimedia), exhaustive reading of textbooks and articles, maximum demand in learning assessment topics, all this and much more granted her pass to enter and remain in the field. Practice fields were opportunities for encounters with social reality, in pediatric hospitals, health centers, residents’ associations and communities. Ana could not resist the opportunities to be with students working in more vulnerable communities. There, in everyday scenes, it was possible to perceive Ana as a citizen, political and academic, scientifically solid and socially committed, to discuss the limits and possibilities for prescriptions/guidelines based on food science and grounded in the economic and social condition of the communities. The teaching of maternal and child nutrition in Bahia was and still is influenced by the teachings of Professor Ana.

In addition to the scientific production and legitimation of the Nutrition area, it is important to point out its contribution in the training of professionals. Over the years that she was in the Graduate Program in Nutrition (*Programa de Pós-Graduação em Nutrição* – PGNUT), she participated intensely in the life of the program, coordinating it for two terms and guiding Master’s (28) and Doctoral (10) students. Ana was also a member of the faculty of the Graduate Program in Collective Health at Universidade Federal da Bahia (*Programa de Pós-Graduação em Saúde Coletiva da Universidade Federal da Bahia* – PPGSC-ISC/UFBA). Her contributions, as well as her intellectual generosity, were valued by faculty and students, who always awaited her precise and respectful comments. In times of frivolity and inconsistency, Ana had intense relationships with those she trusted and remained combative in the face of what she believed to be a space of critical awareness, an expression of a democratic and pluricultural society, a place of respect for the other, to build and disseminate knowledge: the university. It was this academic spirit that, on a daily basis, in more than three decades at ENUFBA, served as an inspiration for many students and co-workers to design their trajectories inspired by Professor Ana.

## CONCLUSION

The scope of the subjects addressed in her scientific production was the result of his restlessness, with the central concern of generating knowledge to face the complex health and nutrition problems in Brazil. With this exercise of revisiting Ana’s academic and professional history, we leave here our sincere thanks to the one who has always valued a Brazilian State that induces economic and social policies capable of promoting the inclusion of excluded layers of society, expanding and ensuring social rights to those populations still marginalized and without the gains arising from the country’s progress and wealth. In addition, the one that has always invested in strengthening universities, research institutes and the apparatus of science and technology. Aware of the budget cuts of the Ministry of Education and Culture (*Ministério da Educação e Cultura* – MEC) and the Ministry of Science, Technology and Innovation (*Ministério de Ciências e Tecnologia e Inovação* – MCTI) observed in the Temer and Bolsonaro administrations, she was concerned with the direction of the new generations of researchers and the support of research.

Finally, in an attempt to express her caring and loving way, we share here with you the impression of feeling her smile, her welcoming look and hearing Ana’s own voice: “Are you ok?”
